# Analysis of brain activity for speech stimuli and child development of an infant with neurosyphilis: case report

**DOI:** 10.1590/2317-1782/20242023303en

**Published:** 2024-09-13

**Authors:** Isabelle Costa de Vasconcelos, Thalita da Silva Oliveira, Ana Beatriz Santos, Mylena Taise Azevedo Lima, Joseli Soares Brazorotto, Aryelly Dayane da Silva Nunes Araujo, Edgard Morya, Sheila Andreoli Balen

**Affiliations:** 1 Programa Associado de Pós-graduação em Fonoaudiologia, Universidade Federal do Rio Grande do Norte – UFRN - Natal (RN), Brasil, Universidade Federal da Paraíba – UFPB - João Pessoa (PB), Brasil, Universidade Estadual de Ciências da Saúde de Alagoas – UNCISAL, Maceió (AL), Brasil.; 2 Unidade de Atenção à Saúde da Criança e Adolescente – UASCA, Hospital Universitário Onofre Lopes – EBSERH - Natal (RN), Brasil.; 3 Laboratório de Inovação Tecnológica em Saúde – LAIS, Universidade Federal do Rio Grande do Norte – UFRN - Natal (RN), Brasil.; 4 Programa de Pós-graduação em Gestão e Inovação em Saúde – PPgGIS, Universidade Federal do Rio Grande do Norte – UFRN - Natal (RN), Brasil.; 5 Departamento de Fonoaudiologia, Universidade Federal do Rio Grande do Norte – UFRN - Natal (RN), Brasil.; 6 Instituto Internacional de Neurociências Edmond e Lily Safra, Instituto Santos Dumont – ISD - Macaíba (RN), Brasil.

**Keywords:** Spectroscopy, Near-Infrared, Newborn Infants, Speech Perception, Auditory Perception, Syphilis, Congenital, Neurosyphilis

## Abstract

Neurosyphilis is an infection of the central nervous system caused by Treponema pallidum and may be symptomatic or asymptomatic in children with congenital syphilis. This study aims to describe the cortical activation pattern of a four-month-old infant with neurosyphilis using functional near-infrared spectroscopy (fNIRS). Born at term weighing 3,475 kg, she presented a Venereal Disease Research Laboratory (VDRL) test of 1:32 and changes in the cerebrospinal fluid test. She underwent treatment with crystalline penicillin for 10 days before discharge from the hospital. In the audiological evaluation, she presented normal tympanometry, otoacoustic emissions evoked by transient stimulus, brainstem auditory evoked potential with click stimulus at 80 and 30 dB nHL bilaterally. The Bayley III Scale was applied to assess language, cognition and motor development, showing delays in expressive language and broad motor skills. In the fNIRS acquisition, data were collected through 20 channels divided between the cerebral hemispheres. The /ba/ and /da/ stimuli were presented at 40 dB HL with the Psychopy software through a headphone. Data analysis used the MNE and MNE-NIRS toolboxes in the Spyder environment. The average by channel, ROI, and condition was exported for analysis. A similar theta coefficient was observed between the conditions and channels evaluated in both cerebral hemispheres, with a greater amplitude of oxyhemoglobin (HbO) being observed in the anterior position when compared to the posterior region of the temporal lobe. Therefore, this case report highlights the need to monitor the child development of babies with neurosyphilis.

## INTRODUCTION

Syphilis is a systemic infectious disease (STI) of chronic evolution, susceptible to outbreaks of exacerbation and periods of latency, and can be transmitted vertically from mother to fetus and/or infant. The incidence of congenital syphilis increased from 700,000 to 1.5 million cases reported annually between 2016 and 2023 worldwide^(1)^. In the analysis of the Global Burden of Disorders, Injuries and Risk Factors Study from 1990 to 2019^(2)^ neonatal disorders were the main cause of burden in this age group (23.0% in 1990 and 32.4% in 2019). Sexually transmitted infections (accounting for congenital syphilis) have seen a modest reduction over the years, being reported among the ten most common causes in this period of life^(2)^.

In Brazil, syphilis continues to be a major public health problem, despite the fact that it is a preventable disease with a relatively low cost of medication for women/pregnant women and is available through the Brazilian Health System (SUS). In this way, its occurrence highlights shortcomings in the notification and management of cases in health services^(3-5)^.

With the mandatory testing of mothers at the time of birth and of newborns immediately after birth, fortunately, the identification of congenital syphilis has been carried out at birth and treated in the national territory^(6)^. However, there are also gaps in the management of these cases throughout the child's development, which makes it impossible to close the case and identify late impacts on the neurodevelopment, hearing and vision of these children^(7)^.

A longitudinal study^(8)^ described 26% of children with congenital syphilis with neurodevelopmental alterations including cerebral palsy, visual impairment, mental disability, microcephaly, sensorineural hearing loss, and language delays. Of these, 36% had language delays without the presence of hearing impairment. There are other reports in the literature of developmental delays in children with congenital syphilis^(6,9,10)^.

Hearing loss is a possible late manifestation of congenital syphilis and is a globally recognized risk indicator for hearing loss (IRDA)^(11)^. Neurosyphilis can also affect the central nervous system (CNS), with varied clinical manifestations, ranging from alterations in the cerebrospinal fluid (CSF) in asymptomatic newborns to more serious symptoms such as progressive cerebral palsy. Around 23% of infants born to mothers with syphilis have neurosyphilis^(12)^. This prevalence is already estimated at 60% according to the Brazilian Protocol for Sexually Transmitted Infections - PCDT- STI^(6)^, based on the presence of alterations in the CSF. Although there is still controversy about the prevalence and incidence of neurosyphilis in children with congenital syphilis, its occurrence is more likely in children born symptomatic than asymptomatic^(6)^. Thus, the diagnosis of neurosyphilis is complex, with CSF evaluation being the only way to diagnose asymptomatic neurosyphilis^(12)^.

Early neurosyphilis manifests soon after a syphilitic infection, can cause meningitis and affect the cranial nerves. Diagnosing neurosyphilis remains a challenge, as there is no gold standard test for its detection. There is no single highly sensitive and specific test for the diagnosis of neurosyphilis; consequently, the diagnosis is based on a combination of clinical findings - similar to the one presented in this case report, CSF alterations and CSF VDRL result. VDRL is the most widely used method, with sensitivity ranging from 50% to 70%^(13)^. In this case report, the infant received treatment. There is a case report in the literature^(14)^ showing alterations on physical examination, with vesiculobullous lesions and a diagnostic suggestion of palmoplantar pemphigus associated with hepatosplenomegaly in a newborn with neurosyphilis without adequate treatment^(14)^.

It is important to emphasize that all cases with a reactive VDRL test in the CSF, regardless of the presence of neurological and/or ocular signs and symptoms, as well as cases with non-reactive VDRL in the CSF, but with biochemical changes in the CSF and the presence of neurological and/or ocular signs and symptoms and/or CNS imaging findings characteristic of the disease, provided that the findings cannot be explained by another disease, should be treated for neurosyphilis^(6)^.

This case report aims to describe the cortical activation pattern of a four-month-old infant with neurosyphilis diagnosed and treated using functional near-infrared spectroscopy (fNIRS). fNIRS allows for the functional assessment of brain activity by calculating variations in the concentration of oxygenated hemoglobin (HbO), deoxygenated hemoglobin (HbR) and total hemoglobin (HbT), which in turn allows for the quantitative and qualitative identification of hemodynamics and neuronal activation^(15-17)^.

An advantage of fNIRS is that it makes it possible to identify the location and specialization of neural responses. With it, you can identify the cortical structures, or group of cortical structures, that mediate selected processes^(15)^. This tool is considered promising for assessing the functioning of children's cerebral cortex and contributes to expanding knowledge about aspects related to neurodevelopment and cognition in children, as well as investigating auditory processing involving stages such as auditory detection and discrimination^(16,18-20)^. It is an effective tool for assessing hearing at the cortical level in children and can be combined with other existing, standardized hearing assessment methods^(18)^.

The report of this case with audiological assessment, brain activity to speech stimuli using fNIRS and child development can contribute to advances in its applicability in the monitoring of infants affected by neurosyphilis.

## CLINICAL CASE PRESENTATION

This is a clinical study, submitted and approved under number 5.323.957 by the Institutional Research Ethics Committee (IREC), have been signed by the responsible parties in the Free and Informed Consent Form, approved by this Committee. The data mining took place at the research laboratory, with the case being received and monitored at the outpatient unit.

The clinical case concerns a four-month-old infant, brought in by an infectious disease specialist for audiological evaluation, as she had not undergone Neonatal Hearing Screening. The baby was born by normal delivery at 39 weeks and two days, weighing 3.475 kg, with a head circumference of 34.5 cm, a length of 51.5 cm, an Apgar score of 9' in the first five minutes. He presented hyperbilirubinemia and underwent phototherapy for three days, VDRL with a titration of 1:32 and a cerebrospinal fluid test with 64 cells, considered altered, since the standard is up to 25 cells. Thus, the baby was diagnosed and treated for neurosyphilis. The mother reported that syphilis was diagnosed at the first prenatal visit, with a VDRL of 1:64; mother and partner were treated with Benzetacil for three weeks. After treatment, maternal VDRL dropped, with a level of 1:32, but remained the same at delivery. Therefore, the mother's and infant's ownership was the same at birth, and the infant had alterations in the cerebrospinal fluid test. No X-rays of the long bones and eye sockets were taken at birth. The eye and heart tests were normal. These data were collected by interviewing the mother before starting the audiological evaluations, and some information was recorded in the child's and pregnant woman's booklet. The infant's first pediatric follow-up appointment at the Pediatrics Unit was only at four months old, and she had a VDRL of 1:16, a test requested by the UBS nurse (SIC).

As for hearing tests, the following procedures were carried out at four months of age, prior to the fNIRS test.

**Figure 1:**
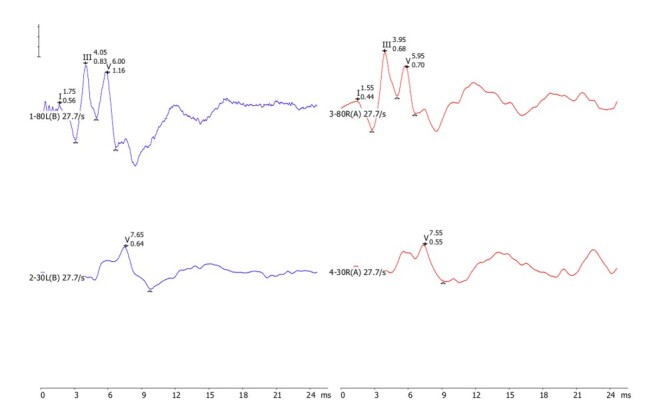
Neural response of the two promediations observed at 80 dB nHL and 30 dB nHL in both ears with the click stimulus (in blue left ear and red right ear) of the BAEP

*Note*: The figure shows the click BAEP response with the sum of the two promediations carried out in each ear and the intensity with waves I, III and V marked at 80 dB nHL and wave V at 30 dB nHL. The numerical values next to each Roman numeral of the wave refer to the latency (ms) (top) and the amplitude (µ) (bottom)

- Otoscopy with the Riester E-Scope 3.7V LED Otoscope, there was no accumulation of cerumen or foreign bodies that prevented the audiological procedures from being carried out;
- Tympanometry with 1,000Hz probe: on the Interacoustic AT235 equipment, tympanometry was performed automatically by inserting a pressure variation in the external acoustic meatus and observing the presence of a positive peak at 15 daPA and 32 daPA, with a volume of 0.47 and 0.42 cc and compliance of 0.85 and 0.62 cc, respectively, in the right and left ears;
- Transient Evoked Otoacoustic Emissions (TEOAE): TEOAEs were collected using the IHS SmartTrOAE module with a 10D otoacoustic emissions (OAE) probe. A 75 µs non-linear click was used, at 80 dB SPL at a 21.1/sec rate and automatic gain control. 1,024 sweeps were presented, in an analysis window of 2.5 to 20 ms, recorded in two buffers. A/B for reproducibility check. A signal-to-noise ratio (SNR) ≥ 6 dB from 1.0 to 4.0 kHz was considered to be present in each frequency band. The result obtained in the infant was the presence of TEOAEs in both ears in the 1.5 to 4.0 Hz frequency bands, with absence in the 1.0 kHz band bilaterally;
- Brainstem Auditory Evoked Potential (BAEP): was carried out on the IHS SmartEP module with insert headphones. The click was displayed at a speed of 27.7/sec. Two rarefied polarity measurements of 2,048 sweeps at 80 dBnHL were carried out, using a 30-3,000 Hz filter and a 24 ms analysis window at 80 dBnHL to observe the presence, latency and amplitude of waves I, III and V and interpeaks I-III, III-V and I-V.
- Following the two promediations, the repeatability of the two recordings was visually examined. Throughout the two premeditations, the repeatability of the two recordings was visually examined. At 30 dBnHL, the V wave was observed bilaterally, with reproducibility from two sequential measurements which, when added together, can be seen on Figure 1.

In addition to the audiological assessment, the infant's development was evaluated using the Bayley-III Scale, in the receptive and expressive language, cognition, large and fine motor sub-scales (Chart 1). During the application, the child was accompanied by the mother and remained calm and cooperative. Table 1 shows that the scaled score on the Bayley-III Scale was below expectations in the areas of expressive language and fine motor skills, while performance on the other subscales was within the average range, although performance on the cognitive and receptive language scales was closer to the below-average range.

**Chart 1:** Scaled score on the Bayley-III Scale for infants with neurosyphilis.

	Scaled Score Profile						
	Cognitive	Receptive Language	Expressive Language	Fine Motor	Gross Motor		
19	.	.	.	.	.	19	
18	.	.	.	.	.	18	
17	.	.	.	.	.	17	
16	.	.	.	.	.	16	Well above average
15	.	.	.	.	.	15	
14	.	.	.	.	.	14	
13	.	.	.	.	.	13	Above average
12	.	.	.	.	X	12	
11	.	.	.	.	.	11	
10	.	.	.	.	.	10	Average
9	.	.	.	.	.	9	
8	X	X	.	.	.	8	
7	.	.	.	.	.	7	Below average
6	.	.	X	.	.	6	
5	.	.	.	X	.	5	
4	.	.	.	.	.	4	Far below average
3	.	.	.	.	.	3	
2	.	.	.	.	.	2	
1	.	.	.	.	.	1	

**Caption:** the mark with an X corresponds to the value obtained with the infant in each subscale

**Table 1:** Latency and amplitude of the BAEP click at 80 dB nHL of the clinical case bilaterally

	I	III	V	I-III	III-V	I-V
Latency (ms)						
Right ear	1.55	3.95	5.95	2.40	2.00	4.40
Left ear	1.75	4.05	6.00	2.30	1.95	4.25
Amplitude (μV)						
Right ear	0.44	0.68	0.70			
Left ear	0.56	0.83	1.16			

**Figure 2:**
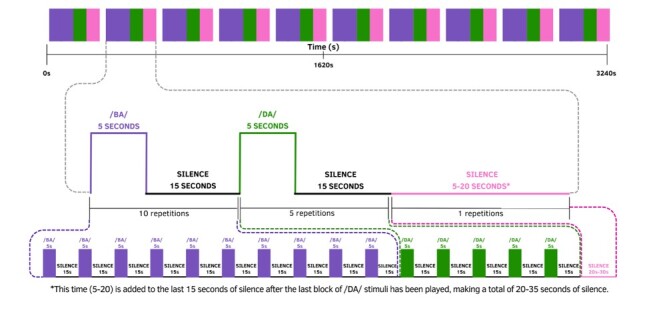
Experimental design of speech stimuli

It was carried out using the NIRStar application for collecting data on the NIRx Medical Tech (760 to 850 nm, 7.81Hz). Natural syllables /ba/ and /da/ recorded by a native Portuguese speaker were presented. The stimuli recordings were provided by Prof. Dr. Pedro de Lemos Menezes, from LATEC-UNCISAL. Each syllable lasted for 300 ms, with an initial rate of 48 kHz.The stimuli were organized and presented using Psychopy, an open-source software written in Python for research in neuroscience.The /ba/ and /da/ audios were edited in the Audacity program (Audacity® Cross-Platform Sound Editor), a open-source tool for digital audio editing, in order to adjust the sampling rate to 44.1 kHz and adapt it to Psychopy's sampling rate.This software enables synchronization between the present stimuli and the concurrent acquisition of functional near-infrared spectroscopy (fNIRS) data, thereby facilitating precise temporal alignment between the two modalities.Figure 2 shows the experimental stimuli design, in which 10 blocks were run for 53 minutes.

**Figure 3:**
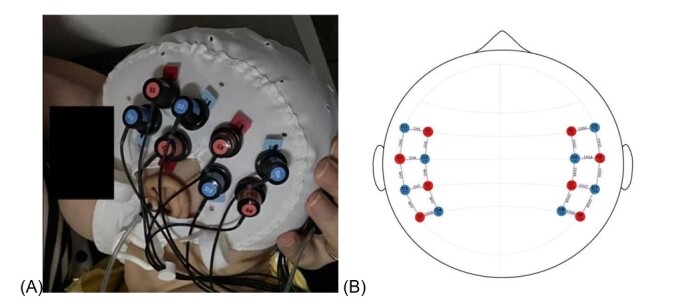
(A) Positioning of the optodes on the infant's head; (B) Arrangement of the optodes in the temporal lobe. In both, red are the emitting optodes, blue the detectors. In B, the purple lines represent the channels formed.

THus, for the current moment, the baby was laid on her mother's lap, in natural sleep. The fNIRS cap was put on, consisting of 16 optodes (8 sources and 8 detectors) forming 20 channels (10 per hemisphere) separated by 3 cm (Figure 3A). All were positioned in the temporal lobe (Figure 3B), with the region of interest (ROI) being anterior, middle and posterior. Before starting the stimuli presentation, EAR-Tone ER 3C insert earphones (10 ohms) were plugged into each external acoustic meatus (Figure 3A).

It was only possible to execute the procedure in full at 40 dBHL, as the patient was uncomfortable at all the above intensities.At this intensity of 40 dBHL, the entire 10-block recording was processed.

The MNE and MNE-NIRS toolboxes for data analysis and visualization^(21)^ were use for data processing, using Spyder IDE, an environment for Python programming in a scientific context..

The methodological procedure for analysis followed an orderly sequence of steps. Thus, the libraries were installed to ensure the process functionality. Next, a dataframe was created for data storage, followed by the import of the raw fNIRS data. A pre-analysis and filtering phase was carried out, starting with verifying the optode position using graphical representations, as well as identifying and renaming the triggers related to the stimuli. The stimuli duration was defined for subsequent analysis using the Generalized Linear Model (GLM), and the raw signal was converted into optical density. The data quality was then assessed by quantifying the coupling quality between the optodes and the scalp per channel (SCI - Scalp coupling index). Channels with good quality were quantified with SCI greater than 0.521. Temporal Derivative Distribution Repair (TDDR) was used to correct artifacts (baseline shift and spikes). The optical density conversion to hemoglobin concentrations was carried out by implementing the modified Beer-Lambert law. Subsequently, filters were applied: from 0.05 to 0.5 Hz, with a high bandwidth of 0.2 and a low bandwidth of 0.02 to restrict the frequency range. The event of interest was extracted and rejection criteria were applied to remove low-quality epochs, after checking which epochs were excluded for each stimulus. Scatter plots were generated to check the coherence of the answers between the types of stimuli and the concentrations of HbO and HbR.

The subsequent stage involved implementing the Generalized Linear Model (GLM) to measure the hemodynamic response. The matrix design was created by modeling expected hemodynamic responses based on the Statistical Parametric Mapping (SPM) model. Response regions of interest (ROI's) were defined (anterior, middle and posterior), and GLM analyses were fitted for all channels (1 to 20) and ROI's. After this procedure, the metric results per channel, ROI's and condition were exported to a .xlsx file for statistical analysis. As this study consists of a clinical case, it was possible to describe in Tables 2 and 3 the theta value (estimated coefficient of the GLM model) of HbO for the conditions (/ba/ and /da/), the regions of interest (ROI) and the channels recorded.

**Table 2:** *Theta* value of the mean of the conditions and ROI of the HbO response of the clinical case

Condition/ ROI	/ba/ *tetha*	/da/ *theta*
Left Hemisphere		
**Anterior**	**10.08**	**11.75**
Medial	7.04	7.21
Posterior	4.13	5.43
Right Hemisphere		
**Anterior**	**12.15**	**10.63**
**Medial**	**12.31**	**10.77**
Posterior	5.23	3.84

**Table 3:** Theta value of the average of the conditions and channels of the HbO response of the clinical case

Condition/ Channels	/ba/ *tetha*	/da/ *tetha*
CH 1	4.25	8.07
**CH 2**	**11.54**	**11.75**
**CH 3**	**14.06**	**15.60**
**CH 4**	**14.65**	**14.61**
CH 5	4.18	3.66
CH 6	4.12	4.95
CH 7	7.99	8.20
CH 8	5.60	5.58
CH 9	0.90	4.21
CH 10	5.96	6.63
CH 11	5.60	5.12
**CH 12**	**11.72**	8.88
**CH 13**	**22.42**	**21.45**
**CH 14**	**21.63**	**21.19**
CH 15	8.54	6.82
CH 16	7.75	6.67
CH 17	9.69	6.18
CH 18	7.19	6.42
CH 19	4.79	3.36
CH 20	4.09	2.06

**Figure 4:**
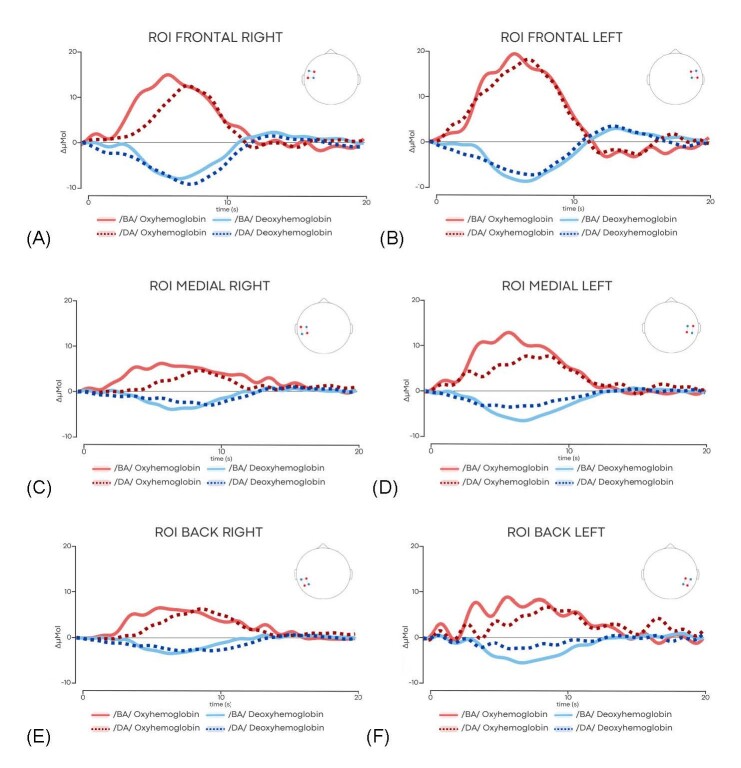
(A) Average HbO (in red) and HbR (in blue) levels of the response to the /BA/ and /DA/ stimuli in the anterior (frontal) ROI on the right; (B) Average HbO (in red) and HbR (in blue) levels in response to /BA/ and /DA/ stimuli in the anterior (frontal) ROI on the left; (C) Average HbO (in red) and HbR (in blue) levels in response to /BA/ and /DA/ stimuli in the medial ROI on the right. (D) Average HbO (in red) and HbR (in blue) levels of the response to the /BA/ and /DA/ stimuli in the medial ROI on the left; (E) Average HbO (in red) and HbR (in blue) levels of the response to the /BA/ and /DA/ stimuli in the posterior ROI (back) on the right; (F) Average HbO (in red) and HbR (in blue) levels of the response to the /BA/ and /DA/ stimuli in the posterior ROI (back) on the left. ΔμMol - micromolar

Table 2 shows that the theta value for both the /ba/ and /da/ stimuli was higher in the anterior ROI of the left hemisphere (HE) and in the anterior and medial ROI of the right hemisphere (HD). In relation to the (CH) channels, higher theta values can be seen in CH2, CH3, CH4 (anterior region of the HE) and CH13, CH12 and CH14 (anterior region of the HD) (Figure 3B, Table 3) of both stimuli. In both ROI and channel analysis, there was no difference in the HbO theta value of the clinical case presented as a function of the stimuli used (Figure 4A-F).

## DISCUSSION

The audiological evaluation showed normality in the peripheral portion of hearing up to the brainstem, as evidenced by the correlation between the audiological tests performed.

A tympanometric curve with a positive peak bilaterally indicates middle ear normality^(22)^. TEOAEs were present except in the 1kHz frequency band bilaterally in the case described. This finding may be related to the lower level of TEOAE response influenced by the smaller middle and outer ear area in infants compared to adults^(23)^. On the other hand, it was observed that infants with a history of congenital infections have a lower level of response at 1.5kHz, although this was not statistically significant^(24)^. There were also no differences in the inhibitory effect of TEOAEs in infants with congenital syphilis when compared to control infants^(25)^, an assessment that was not carried out in this case report.

The BAEP responses clicked at 80 dB nHL showed wave I, III and V latencies within the expected range, with interpeaks I-III, III-V and I-V, showing maturation of the central auditory pathway^(26,27)^. There were no interaural differences in wave V between the ears (Table 1). These findings correlate with those found^(28)^ in infants with congenital syphilis and controls, performed on the same equipment. The same was observed in primary studies^(8,25,29)^ and in a systematic review^(30)^ when no differences were found in click BAEP responses in neonates/children with congenital syphilis.

Although congenital syphilis is a highly prevalent risk indicator, a retrospective study^(31)^ found that there was no confirmation of hearing impairment in all children aged zero to three with this history assessed at a Hearing Health Service of the Brazilian National Health System (SUS) between 2011 and 2019. When analyzing the^(32)^ occurrences of risk indicators and the outcomes of Neonatal Hearing Screening (NHS) in a public maternity hospital from 2019 to 2021, it was found that congenital syphilis was the ninth most prevalent indicator, however, in isolation, it did not represent a risk for hearing impairment, data that corroborates the findings in this case study.

It should be noted that since 1986^(33)^ all pregnant women have been tested for HIV and syphilis at the time of delivery and, when positive, their newborns are also evaluated. Once the results are positive for syphilis, the mother-infant dyad is placed on perinatal treatment for ten days before being discharged from hospital. Thus, the absence of audiological alterations observed in this case study confirms the positive effects of this public policy in Brazil.

However, hearing alterations in congenital syphilis do not necessarily manifest themselves in the first few months of life, but can appear throughout childhood and into adulthood^(6)^. Therefore, it is essential to establish a six-monthly follow-up for infants affected by congenital syphilis, in order to identify hearing and developmental alterations as early as possible^(6)^.

The investigation of the auditory pathway in a more comprehensive way, as well as cortical activation for speech stimuli is relevant in this population^(7)^, particularly in cases with other impairments, such as neurosyphilis, because the bacterial infection affects the central nervous system^(6)^. In disorders that can have a variety of impacts on childhood, it is also essential to monitor child development. In cases of treated congenital syphilis, performance on the Bayley-III scale in the first months of life was similar to that of infants in the control group of the same age, showing that most subjects performed at or above the average on the Bayley subscales^(34)^. Nevertheless, the results found in this case report call attention to the below-average performance in expressive language and fine motor skills, and borderline performance in cognition and receptive language, which must be taken into account since this is a case of neurosyphilis, indicating the need for family guidance and follow-up.

At the time of the evaluation at 4 months of age, there was an impact on the infant's development, but due to the design of the case report, it is challenging to conclude whether this impact refers directly to the syphilis infection, or whether it is also related to other perinatal indicators such as hyperbilirubinemia or socioeconomic issues.

As for the fNIRS responses with speech stimuli, in this clinical case it can be seen that there was a change in cerebral hemodynamics characterized by the phase difference and the temporal correlation between the HbO and HbR^(35)^ time series both in the presence of /ba/ and /da/ stimuli, showing the auditory detection of this four-month-old infant with neurosyphilis.

There was a similar HbO response to both stimuli, with a higher theta value in both hemispheres' anterior ROI and the HD's medial ROI. The channels with the greatest amplitude in theta value were those in the anterior region of the temporal lobe in both hemispheres. In the case described, the infant had no difference between the responses observed between the stimuli, but it is clear that she detected the speech sounds.

In the clinical case presented, there was no lateralization of the response to the left hemisphere, which differs from other studies^(16,36)^, which show greater lateralization of responses to the left hemisphere. In addition, there was a predominantly anterior activation of the temporal lobe and bilateral activation found in this case report, which also differs from research that has shown a greater extent of activation in the temporal cortical region of the brain.

However, it is important to note that these studies were conducted in populations without risk indicators for hearing loss^(19,36-39)^. Therefore, it is possible to infer that the fNIRS results found in this case may be related to the involvement of neurosyphilis, as well as the neurodevelopmental impacts observed. Thus, it is necessary to study new clinical cases to determine whether the pattern found in this case report could be the pattern related to neurosyphilis or a variation of typical responses, since fNIRS studies in this population are still incipient.

fNIRS can be an important tool for monitoring the functional organization of the infant brain during development, as it measures the attenuation of light by brain tissues due to changes in the concentration of oxygenated hemoglobin (HbO) and deoxygenated hemoglobin (HbR) within the recorded region. These changes reflect the vascular response associated with neural activity and thus represent a measure of the brain's functional activation^(35)^. Functional connectivity even in a resting state, as in the fNIRS recording with speech stimuli in this case report, when the infant was in natural sleep, can be observed by the intrinsic functional organization of the brain^(35)^. Therefore, the changes observed in HbO and HbR during the presence and absence of auditory stimuli during sleep show that there was an auditory detection response.

The limitations of this study include the fact that it was a single case study, since in general, neuroimaging techniques in developing populations can have influences and variations such as the presence of movement artifacts, short recording durations and small sample size, which makes it difficult to generalize expected patterns by age group in this type of procedure^(19)^.

Thus, the case report presented illustrates a pattern of results for which continued follow-up will be necessary to identify whether developmental changes will still occur in subsequent evaluations and whether they correlate with the pattern of neuroimaging responses (fNIRS).

The impossibility of longitudinal monitoring of the child, since the family has various social vulnerability issues and has significant difficulties in traveling to face-to-face medical, audiological and neurodevelopmental reassessments, has reduced the possibility of follow-up observation, which is known to be necessary for this population.

Because of the family's vulnerability, it is understood that the child may be exposed to other Social Determinants of Health that can also interfere with their development, which is another factor that reinforces the need for this follow-up/monitoring. This is another issue often related to syphilis in pregnant women and evidenced in the possible consequences generated by congenital syphilis, including neurosyphilis. A study in the region of Tocantins^(12)^ found 29 cases of neonates with neurosyphilis, most of whom were asymptomatic and had low socioeconomic status and lower maternal schooling.

There is evidence in the literature pointing to delayed neuropsychomotor development in children with congenital syphilis and neurosyphilis^(6,9,10)^. As far as audiological aspects are concerned, in addition to specialist care in the hearing health care network, as provided for in international and national guidelines^(11,40)^, developmental aspects should be monitored in Primary Health Care, with a referral for specialist assessment when the need is identified^(40)^. As for the other developmental domains, especially cognitive, language and motor, this case highlighted the possibility of these impacts which should be observed throughout the development of these infants for early identification and intervention. Thus, with the diagnosis of neurosyphilis, this surveillance and integration at all levels of health care becomes even more urgent, and multidisciplinary monitoring is essential.

It is necessary to expand the study of the interaction between syphilis and possible neurodevelopmental alterations, thus predicting delays in their language and cognitive development, thereby improving measures for the identification, diagnosis and early intervention of alterations in the neurodevelopment of these infants, as well as contributing to the improvement of public policies related to follow-up in the Maternal and Child Care Network in the first years of life.

## CONCLUSION

The description of this clinical case showed a change in cerebral hemodynamics in the presence of speech stimuli, characterized by greater activation in the anterior region of the temporal lobe of both cerebral hemispheres. There was a delay in the development of the motor and expressive language domains. These findings emphasize the importance of the Comprehensive Care Network for infants with congenital syphilis involving audiological monitoring and child development.
