# Supplementary material for: Analysis of brain activity for speech stimuli and child development of an infant with neurosyphilis: case report
Source: Codas. 2024 Sep 13;36(5):e20230303. doi: 10.1590/2317-1782/20242023303en (PMC11404838; doi:10.1590/2317-1782/20242023303en)
Supplement: Translation

# Translation: Análise da atividade cerebral para estímulos de fala e desenvolvimento infantil de um lactente com neurossífilis: relato de caso

**DOI:** 10.1590/2317-1782/20242023303pt

**Authors:** Isabelle Costa de Vasconcelos, Thalita da Silva Oliveira, Ana Beatriz Santos, Mylena Taise Azevedo Lima, Joseli Soares Brazorotto, Aryelly Dayane da Silva Nunes Araujo, Edgard Morya, Sheila Andreoli Balen

**Affiliations:** 1 Programa Associado de Pós-graduação em Fonoaudiologia, Universidade Federal do Rio Grande do Norte – UFRN - Natal (RN), Brasil, Universidade Federal da Paraíba – UFPB - João Pessoa (PB), Brasil, Universidade Estadual de Ciências da Saúde de Alagoas – UNCISAL, Maceió (AL), Brasil.; 2 Unidade de Atenção à Saúde da Criança e Adolescente – UASCA, Hospital Universitário Onofre Lopes – EBSERH - Natal (RN), Brasil.; 3 Laboratório de Inovação Tecnológica em Saúde – LAIS, Universidade Federal do Rio Grande do Norte – UFRN - Natal (RN), Brasil.; 4 Programa de Pós-graduação em Gestão e Inovação em Saúde – PPgGIS, Universidade Federal do Rio Grande do Norte – UFRN - Natal (RN), Brasil.; 5 Departamento de Fonoaudiologia, Universidade Federal do Rio Grande do Norte – UFRN - Natal (RN), Brasil.; 6 Instituto Internacional de Neurociências Edmond e Lily Safra, Instituto Santos Dumont – ISD - Macaíba (RN), Brasil.

**Keywords:** Espectroscopia de Luz Próxima ao Infravermelho, Neonato, Percepção de Fala, Percepção Auditiva, Sífilis Congênita, Neurossífilis

## Abstract

A neurossífilis é uma infecção do sistema nervoso central causada pelo Treponema pallidum, podendo ser sintomática ou assintomática nas crianças com sífilis congênita. Este estudo visa descrever o padrão de ativação cortical de uma lactente de quatro meses com neurossífilis utilizando o funcional near-infrared spectroscopy (fNIRS). Nascida a termo com 3.475 Kg, apresentou teste Venereal Disease Research Laboratory (VDRL) de 1:32 e alteração no exame de líquor cefalorraquidiano. Realizou tratamento com penicilina cristalina por 10 dias antes da alta hospitalar. Na avaliação audiológica apresentou normalidade na timpanometria, emissões otoacústicas evocadas por estímulo transiente, potencial evocado auditivo de tronco encefálico com estímulo clique a 80 e 30 dB nNA bilateralmente. Foi aplicada a Escala Bayley III para a avaliação do desenvolvimento de linguagem, cognição e motor, apresentando atrasos na linguagem expressiva e no motor amplo. Na aquisição do fNIRS os dados foram coletados por 20 canais divididos entre os hemisférios cerebrais. Os estímulos /ba/ e /da/ foram apresentados a 40 dB NA com o auxílio do programa Psychopy por um fone de ouvido. A análise dos dados utilizou as toolboxes MNE e MNE-NIRS no ambiente Spyder. A média por canal, ROI e condição foi exportada para análise. Observou-se um coeficiente theta similar entre as condições e canais avaliados de ambos os hemisférios cerebrais, sendo observado maior amplitude da oxihemoglobina (HbO) na posição anterior quando comparados a região posterior do lobo temporal. Desta forma, este relato de caso evidencia a necessidade de monitoramento do desenvolvimento infantil de lactentes com neurossífilis.

## INTRODUÇÃO

A sífilis é uma doença infectocontagiosa sistêmica (IST) de evolução crônica, sujeita a surtos de agudização e períodos de latência podendo ser transmitida de forma vertical da mãe para o feto e/ou lactente. A incidência de sífilis congênita aumentou de 700.000 a 1,5 milhão de casos notificados anualmente entre 2016 e 2023 em todo o mundo^(1)^. Na análise do *Global Burden of Disorders, Injuries and Risk Factors Study de 1990 a 2019* ^(2)^ os distúrbios neonatais foram a principal causa de sobrecarga nesta faixa etária (23,0% em 1990 e 32,4% em 2019). As infecções sexualmente transmissíveis (contabilizada pela sífilis congênita) tiveram redução modesta ao longo dos anos, sendo reportada entre as dez causas mais comuns neste período da vida^(2)^.

No Brasil, a sífilis continua sendo um grande problema de saúde pública, apesar de ser uma doença evitável com custo de medicação à mulher/gestante relativamente baixo e disponível no Sistema Único de Saúde (SUS). Desta forma, sua ocorrência evidencia falhas na notificação e gestão dos casos nos serviços de saúde^(3-5)^.

Com a obrigatoriedade da testagem materna no momento do parto e do recém-nascido logo após o nascimento, felizmente a identificação da sífilis congênita tem sido realizada ao nascimento e tratada no território nacional^(6)^. No entanto, há também lacunas na gestão destes casos ao longo do desenvolvimento da criança, o que impossibilita o fechamento do caso e a identificação de impactos tardios no neurodesenvolvimento, na audição e visão destas crianças^(7)^.

Um estudo longitudinal^(8)^ descreveu 26% de crianças com sífilis congênita com alterações no neurodesenvolvimento incluindo paralisia cerebral, deficiência visual, deficiência mental, microcefalia, perda auditiva sensorioneural e atrasos de linguagem. Dessas 36% apresentaram atrasos de linguagem sem a presença de deficiência auditiva. Há outros relatos na literatura da presença de atrasos no desenvolvimento de crianças com sífilis congênita^(6,9,10)^.

A deficiência auditiva é uma possibilidade de manifestação tardia da sífilis congênita, sendo um indicador de risco para a deficiência auditiva (IRDA) reconhecido mundialmente^(11)^. Também pode acometer o sistema nervoso central (SNC), a neurossífilis, com manifestações clínicas variadas, que vão desde alterações do líquido cefalorraquidiano (LCR) em recém-nascidos assintomáticos até sintomatologias mais graves, como paralisia cerebral progressiva. Cerca de 23% dos lactentes nascidos de mães com sífilis têm neurossífilis^(12)^. Esta prevalência já é exposta como 60% segundo o Protocolo Clínico de Doenças Sexualmente Transmissíveis - PCDT- IST^(6)^, com base na presença de alterações no LCR. Embora haja ainda controvérsias sobre a prevalência e incidência da neurossífilis na criança com sífilis congênita, sua ocorrência é mais provável em crianças que nascem sintomáticas do que assintomáticas^(6)^. Assim, o diagnóstico da neurossífilis é complexo, sendo que a avaliação do LCR a única forma de diagnosticar a neurossífilis assintomática^(12)^.

A neurossífilis precoce se manifesta logo após uma infecção sifilítica, pode causar meningite e afetar os nervos cranianos. O diagnóstico da neurossífilis continua a ser um desafio, uma vez que não existe um teste padrão-ouro para sua detecção. Não há um único teste altamente sensível e específico para o diagnóstico de neurossífilis; consequentemente, o diagnóstico é baseado em uma combinação de achados clínicos - semelhante ao apresentado nesse relato de caso, alterações do LCR e resultado do VDRL no LCR. O VDRL é o método mais utilizado, sua sensibilidade varia de 50% a 70%^(13)^. No presente relato de caso, a lactente recebeu tratamento. Há na literatura relato de caso^(14)^ que demonstra alterações ao exame físico, com lesões vesico-bolhosas e sugestão diagnóstica de pênfigo palmoplantar associado a hepatoesplenomegalia em recém nascido com neurossífilis sem tratamento adequado^(14)^.

É importante ressaltar que todos os casos com teste de reagente VDRL no LCR, independentemente da presença de sinais e sintomas neurológicos e/ou oculares, bem como casos com VDRL não reagente no LCR, mas com alterações bioquímicas no LCR e a presença de sinais e sintomas neurológicos e/ou oculares e/ou achados de imagem do SNC característicos da doença, desde que os achados não possam ser explicados por outra doença, devem ser tratados para neurossífilis^(6)^.

Este relato de caso visa descrever o padrão de ativação cortical de uma lactente de quatro meses com neurossífilis diagnosticada e tratada utilizando o *functional near-infrared spectroscopy* (fNIRS). O fNIRS permite a avaliação funcional da atividade cerebral através do cálculo das variações na concentração de hemoglobina oxigenada (HbO), hemoglobina desoxigenada (HbR) e de hemoglobina total (HbT), o que permite, por sua vez, identificar de forma quantitativa e qualitativa a hemodinâmica e a ativação neuronal^(15-17)^.

Uma vantagem do fNIRS, é que ele permite identificar a localização e a especialização das respostas neurais. Com ele, podem ser identificadas as estruturas corticais, ou grupo de estruturas corticais, que medeiam processos selecionados^(15)^. Essa ferramenta é considerada promissora para a avaliação do funcionamento do córtex cerebral infantil e contribui para ampliar o conhecimento sobre os aspectos relacionados ao neurodesenvolvimento e cognição em crianças, além da investigação do processamento auditivo envolvendo etapas como a detecção e discriminação auditiva^(16,18-20)^. É uma ferramenta eficaz para avaliação da audição a nível cortical em crianças, podendo ser associada a outros métodos já existentes e padronizados de avaliação auditiva^(18)^.

Assim, considera-se que o relato deste caso com a avaliação audiológica, da atividade cerebral para os estímulos de fala por meio do fNIRS e do desenvolvimento infantil podem contribuir para os avanços em sua aplicabilidade no acompanhamento de lactentes afetados pela neurossífilis.

## APRESENTAÇÃO DO CASO CLÍNICO

Caracteriza-se de um estudo clínico, submetido e aprovado sob o número n. 5.323.957, pelo Comitê de Ética em Pesquisa da Instituição, tendo a assinatura dos responsáveis do Termo de Consentimento Livre e Esclarecido, aprovado por este Comitê. A coleta dos dados ocorreu no laboratório de pesquisa, com acolhimento e início do seguimento do caso na unidade ambulatorial.

O caso clínico é de uma lactente de quatro meses, encaminhada pela médica infectologista para avaliação audiológica, pois não havia realizado a Triagem Auditiva Neonatal. Nasceu de parto normal com 39 semanas e dois dias, 3.475 kg, 34,5 cm de perímetro cefálico, 51,5 cm de comprimento, Apgar 9’ no primeiro e quinto minutos. Apresentou hiperbilirrubinemia, realizando fototerapia por três dias, VDRL com titularidade de 1:32 e exame de líquor cefalorraquidiano com 64 células, considerado alterado, visto que o padrão é de até 25 células. Realizou tratamento com penicilina cristalina por 10 dias antes da alta hospitalar. Assim, foi diagnosticada e tratada como neurossífilis. A mãe referiu que o diagnóstico da sífilis ocorreu na primeira consulta pré-natal, com VDRL de 1:64; mãe e parceiro receberam tratamento com Benzetacil por três semanas. Após o tratamento, o VDRL materno apresentou queda, com titularidade de 1:32, porém manteve-se no parto. Portanto, a titularidade da mãe e da lactente foi a mesma do nascimento, sendo que a lactente apresentou alterações no exame de líquor cefalorraquidiano. Não foi realizado raio-X de ossos longos e fundo de olho ao nascimento. O teste do olhinho e do coraçãozinho foram normais. Estes dados foram coletados por entrevista com a mãe antes de iniciar as avaliações audiológicas, sendo que algumas informações estavam registradas na caderneta da criança e da gestante. A primeira consulta de pediatria para o seguimento da lactente na Unidade de Pediatria aconteceu somente com quatro meses, sendo que ela estava com VDRL 1:16, exame este, solicitado pela enfermeira da UBS (SIC).

Quanto à avaliação da audição, foram realizados os seguintes procedimentos, aos quatro meses de idade, previamente a avaliação com o fNIRS.

- Meatoscopia: com o Otoscópio LED E-Scope 3,7V *Riester*, não foi constatado acúmulo de cerúmen ou corpos estranhos que impedissem a realização dos procedimentos audiológicos;
- Timpanometria com sonda de 1.000Hz: no equipamento AT235 da *Interacoustic* foi realizada a timpanometria de forma automática inserindo variação de pressão no meato acústico externo e observando a presença de pico positivo em 15 daPA e 32 daPA, com volume de 0.47 e 0.42 cc e complacência de 0.85 e 0.62 cc, respectivamente, nas orelhas direita e esquerda;
- Emissões Otoacústicas Evocadas por estímulo Transiente (EOAT): a coleta das EOAT foi realizada no módulo IHS SmartTrOAE, com sonda de emissões otoacústicas (EOA) 10D. Foi utilizado um clique não linear de 75 µs, a 80 dB NPS a uma taxa de 21,1/seg e controle automático de ganho. Foram apresentados 1.024 *sweeps*, em uma janela de análise de 2,5 a 20 ms, registrados em dois *buffers*. A/B para verificação de reprodutibilidade. Foi considerada presença de resposta por banda de frequência a relação sinal-ruído (SNR) ≥ 6 dB de 1.0 a 4.0 kHz. O resultado obtido na lactente foi de presença das EOAT em ambas as orelhas nas bandas de frequência de 1.5 a 4.0 Hz com ausência na banda de 1.0 kHz bilateralmente;
- Potencial Evocado Auditivo de Tronco Encefálico (PEATE): foi realizado no módulo IHS SmartEP com fones de inserção. Foi apresentado o clique na velocidade de 27,7/seg. Foram realizadas duas promediações na polaridade rarefeita de 2.048 *sweeps* a 80 dBnNA, utilizando filtro de 30-3.000 Hz e janela de análise de 24 ms a 80 dB nNA para observar a presença, latência e amplitude das ondas I, III e V e interpicos I-III, III-V e I-V. Após a realização das duas promediações foi analisada visualmente a reprodutibilidade dos dois registros. Na sequência foram somados para a marcação da latência e amplitude de cada onda (Tabela 1). Na intensidade de 30 dBnNA foi observada a presença da onda V bilateralmente com reprodutibilidade a partir de duas promediações sequenciais que somadas podem ser visualizadas na Figura 1.

**Tabela 1:** Latência e amplitude do PEATE clique em 80 dB nNA do caso clínico bilateralmente

	I	III	V	I-III	III-V	I-V
**Latência (ms)**						
Orelha direita	1.55	3.95	5.95	2.40	2.00	4.40
Orelha esquerda	1.75	4.05	6.00	2.30	1.95	4.25
**Amplitude (μV)**						
Orelha direita	0.44	0.68	0.70			
Orelha esquerda	0.56	0.83	1.16

**Figura 1:** Resposta neural das duas promediações constatadas a 80 dB nNA e 30 dB nNA em ambas as orelhas com estímulo clique (em azul orelha esquerda e em vermelho orelha direita) do PEATE

Nota: Na figura são visualizadas a resposta do PEATE clique com a somatória das duas promediações realizadas em cada orelha e intensidade com marcação das ondas I, III e V em 80 dB nNA e da onda V em 30 dB nNA. Os valores numéricos ao lado de cada número romano da onda referem-se à latência (ms) (parte superior) e a amplitude (µ) (na parte inferior)

Além da avaliação audiológica, o desenvolvimento da lactente foi avaliado por meio da Escala Bayley-III, nas subescalas de linguagem receptiva e expressiva, cognição, motor amplo e fino. Durante a aplicação a lactente estava acompanhada da sua mãe, permanecendo tranquila e colaborativa. Observa-se no Quadro 1, que a pontuação escalonada da Escala Bayley-III esteve abaixo do esperado para as áreas de linguagem expressiva e motor fino, sendo o desempenho nas demais subescalas dentro da média, embora nas escalas cognitiva e linguagem receptiva o desempenho esteja mais próximo da classificação abaixo da média.

**Quadro 1:** Pontuação escalonada na Escala Bayley-III da lactente com neurossífilis

	Perfil de Pontuação Escalonada						
	Cognitiva	Linguagem Receptiva	Linguagem Expressiva	Motor Fino	Motor Grosso		
19	.	.	.	.	.	19	
18	.	.	.	.	.	18	
17	.	.	.	.	.	17	
16	.	.	.	.	.	16	Muito acima da média
15	.	.	.	.	.	15	
14	.	.	.	.	.	14	
13	.	.	.	.	.	13	Acima da média
12	.	.	.	.	X	12	
11	.	.	.	.	.	11	
10	.	.	.	.	.	10	Média
9	.	.	.	.	.	9	
8	X	X	.	.	.	8	
7	.	.	.	.	.	7	Abaixo da média
6	.	.	X	.	.	6	
5	.	.	.	X	.	5	
4	.	.	.	.	.	4	Muito abaixo da média
3	.	.	.	.	.	3	
2	.	.	.	.	.	2	
1	.	.	.	.	.	1	

**Legenda:** a marcação com X corresponde ao valor obtido com a lactente em cada subescala

Em relação ao fNIRS com estímulo de fala, o mesmo foi realizado no NIRx Medical Tech (760 a 850 nm, 7.81Hz) com o programa NIRStar para aquisição de dados. Foram apresentadas sílabas naturais /ba/ e /da/ gravadas por um falante nativo de português. As gravações dos estímulos foram cedidas pelo Prof. Dr. Pedro de Lemos Menezes, do LATEC-UNCISAL. A duração de cada sílaba foi de 300 ms e taxa de 48kHz originalmente. Os estímulos foram organizados e apresentados através do *Psychopy,* um *software* de código aberto escrito na linguagem de programação *Python* voltado para uso em pesquisas em neurociências. Os áudios de /ba/ e /da/ foram editados no programa *Audacity* (*Audacity® Cross-Platform Sound Editor*), um *software* livre voltado para edição digital de áudio, com finalidade de ajustar a taxa de amostragem para 44,1 kHz adequando-o à taxa de amostragem do *Psychopy*. Esse *software* garante a sincronização entre a apresentação dos estímulos com o registro simultâneo dos dados do fNIRS. Na Figura 2 observa-se o design experimental dos estímulos, sendo que foram executados 10 blocos totalizando um tempo de duração de 53 minutos.

**Figura 2:** Design experimental dos estímulos de fala

No momento do registro a lactente foi posicionada deitada no colo de sua mãe, em sono natural. Foi colocada a touca do fNIRS, composta por 16 optodos (8 fontes e 8 detectores) formando 20 canais (10 por hemisfério) separados por 3 cm (Figura 3A). Todos foram posicionados no lobo temporal (Figura 3B), tendo a região de interesse (ROI) anterior, média e posterior. Antes de iniciar a apresentação dos estímulos, os fones de inserção EAR-Tone ER 3C (10 ohms) foram inseridos em cada meato acústico externo (Figura 3A).

**Figura 3:** (A) Posicionamento dos optodos na cabeça da lactente; (B) Arranjo dos optodos no lobo temporal. Em ambas, o vermelho são os optodos emissores, em azul os detectores. No B as linhas roxas representam os canais formados

Apenas foi possível executar o procedimento de forma integral a 40 dBNA, pois para todas as intensidades acima constatou-se incômodo da lactente. Nesta intensidade, de 40 dBNA foi realizado todo o registro contendo os 10 blocos.

Na análise dos dados foram utilizadas as *toolboxes* MNE e MNE-NIRS voltadas para a análise e visualização de dados^(21)^ com auxílio do *Spyder*​​, um ambiente para programação e execução científica na linguagem *Python*.

O procedimento metodológico para análise seguiu uma sequência ordenada de etapas. Inicialmente, as bibliotecas apropriadas foram instaladas para assegurar a funcionalidade do processo. Em seguida, um *dataframe* foi criado para a armazenagem dos dados, seguido pela importação dos dados brutos do fNIRS. Uma fase de pré-análise e aplicação de filtros foi executada, iniciando pela verificação da posição dos optodos por meio de representações gráficas, bem como, identificação e renomeação dos *triggers* relacionados aos estímulos. A definição do tempo de duração dos estímulos foi realizada visando sua posterior análise por meio do Modelo Linear Generalizado (GLM), e realizada a conversão do sinal bruto em densidade óptica. Avaliou-se, então, a qualidade do dado, quantificando a qualidade de acoplamento entre os optodos e o couro cabeludo por canal (SCI - *Scalp coupling index*). Canais com boa qualidade foram quantificados com SCI superiores a 0.5^(21)^. O *Temporal Derivative Distribution Repair* (TDDR) foi utilizado para correção de artefatos (mudança da linha de base e espículas). A conversão da densidade óptica para concentrações de hemoglobina foi realizada pela implementação da lei de *Beer-Lambert* modificada. Posteriormente, foi realizada a aplicação de filtros: de 0.05 a 0.5 Hz, com *high bandwidth* de 0.2 e *low bandwidth* de 0.02 para restringir a faixa de frequência. A extração de eventos de interesse e a aplicação de critérios de rejeição para remover épocas de baixa qualidade foram conduzidas, sendo então verificadas quais épocas foram excluídas para cada estímulo. *Plots* das épocas foram gerados para verificar a coerência das respostas entre os tipos de estímulos e as concentrações de HbO e HbR.

O estágio subsequente envolveu a implementação do Modelo Linear Generalizado (GLM) para modelar a resposta hemodinâmica. A criação do design da matriz foi conduzida, modelando respostas hemodinâmicas esperadas baseadas no modelo *Statistical Parametric Mapping* (SPM). Regiões de interesse de resposta (ROI's) foram definidas (anterior, média e posterior), e análises GLM foram ajustadas para todos os canais (1 a 20) e ROI's. Após esse procedimento, os resultados métricos por canal, ROI's e condição foram exportados para arquivo .xlsx para realização da análise estatística. Como este estudo consta de um caso clínico, foi possível descrever nas Tabelas 2 e 3 o valor de *theta* (coeficiente estimado do modelo GLM) de HbO para as condições (/ba/ e /da/), as regiões de interesse (ROI) e os canais registrados.

**Tabela 2:** Valor de *theta* da média das condições e ROI da resposta de HbO do caso clínico

Condição/ ROI	/ba/ *tetha*	/da/ *theta*
Hemisfério Esquerdo		
**Anterior**	**10,08**	**11,75**
Medial	7,04	7,21
Posterior	4,13	5,43
Hemisfério Direito		
**Anterior**	**12,15**	**10,63**
**Medial**	**12,31**	**10,77**
Posterior	5,23	3,84

Observa-se na Tabela 2 que o valor de *theta* tanto para o estímulo /ba/ quanto /da/ foram mais elevados no ROI anterior do hemisfério esquerdo (HE) e no ROI anterior e medial do hemisfério direito (HD). Em relação aos canais (CH) pode-se observar valores mais elevados de *theta* no CH2, CH3, CH4 (região anterior do HE) e CH13, CH12 e CH14 (região anterior do HD) (Figura 3B, Tabela 3) de ambos os estímulos. Tanto na análise do ROI quanto de canal não foi evidenciada diferença no valor de *theta* do HbO do caso clínico apresentado em função dos estímulos utilizados (Figura 4A-F).

**Tabela 3:** Valor do *theta* da média das condições e canais da resposta de HbO do caso clínico

Condição/ Canais	/ba/ *tetha*	/da/ *tetha*
CH 1	4,25	8,07
**CH 2**	**11,54**	**11,75**
**CH 3**	**14,06**	**15,60**
**CH 4**	**14,65**	**14,61**
CH 5	4,18	3,66
CH 6	4,12	4,95
CH 7	7,99	8,20
CH 8	5,60	5,58
CH 9	0,90	4,21
CH 10	5,96	6,63
CH 11	5,60	5,12
**CH 12**	**11,72**	8,88
**CH 13**	**22,42**	**21,45**
**CH 14**	**21,63**	**21,19**
CH 15	8,54	6,82
CH 16	7,75	6,67
CH 17	9,69	6,18
CH 18	7,19	6,42
CH 19	4,79	3,36
CH 20	4,09	2,06

**Figura 4:** (A) Média dos níveis de HbO (em vermelho) e HbR (em azul) das resposta dos estímulos /BA/ e /DA/ no ROI anterior (frontal) à direita; (B) Média dos níveis de HbO (em vermelho) e HbR (em azul) das resposta dos estímulos /BA/ e /DA/ no ROI anterior (frontal) à esquerda; (C) Média dos níveis de HbO (em vermelho) e HbR (em azul) das resposta dos estímulos /BA/ e /DA/ no ROI medial à direita; (D) Média dos níveis de HbO (em vermelho) e HbR (em azul) das resposta dos estímulos /BA/ e /DA/ no ROI medial à esquerda; (E) Média dos níveis de HbO (em vermelho) e HbR (em azul) das resposta dos estímulos /BA/ e /DA/ no ROI posterior (back) à direita; (F) Média dos níveis de HbO (em vermelho) e HbR (em azul) das resposta dos estímulos /BA/ e /DA/ no ROI posterior (back) à esquerda. ΔμMol - micromolar

## DISCUSSÃO

Foi observado na avaliação audiológica normalidade na porção periférica da audição até o tronco encefálico, sendo evidenciado pela correlação entre os testes audiológicos realizados.

A curva timpanométrica com pico positivo bilateralmente indica normalidade da orelha média^(22)^. No caso descrito houve presença de EOAT exceto na banda de frequência de 1kHz bilateralmente. Este achado pode estar relacionado ao nível de resposta mais baixa das EOAT influenciado pela menor área da orelha média e externa nos lactentes em relação ao adulto^(23)^. Por outro lado, foi observado que lactentes com histórico de infecções congênitas têm menor nível de resposta na frequência de 1.5kHz, porém não estatisticamente significante^(24)^. Também não foram constatadas diferenças no efeito inibitório das EOAT em lactentes com sífilis congênita quando comparados aos lactentes controles^(25)^, avaliação esta que não foi realizada neste relato de caso.

Em relação às respostas do PEATE clique a 80 dB nNA observou-se latências das ondas I, III e V dentro do esperado com interpicos I-III, III-V e I-V, evidenciando processos maturacionais da via auditiva central^(26,27)^. Não foram observadas diferença interaural da onda V entre as orelhas (Tabela 1). Estes achados correlacionam-se aos encontrados^(28)^ em lactentes com sífilis congênita e controles, executados no mesmo equipamento. O mesmo foi observado nos estudos primários^(8,25,29)^ e em uma revisão sistemática^(30)^ quando não foram constatadas diferenças nas respostas do PEATE clique em neonatos/crianças com sífilis congênita.

Embora a sífilis congênita seja um um indicador de risco altamente prevalente, um estudo retrospectivo^(31)^ constatou que não houve confirmação de deficiência auditiva em todas as crianças de zero a três anos com este histórico avaliadas num Serviço de Saúde Auditiva do Sistema Único de Saúde, no período de 2011 a 2019. Ao analisar as^(32)^ ocorrências dos indicadores de risco e os desfechos da Triagem Auditiva Neonatal (TAN) de uma maternidade pública no período de 2019 a 2021, constatou-se que a sífilis congênita foi o nono indicador mais prevalente, porém, de forma isolada, não representou risco para a deficiência auditiva, dados que corroboram os achados neste estudo de caso.

Destaca-se que desde 1986^(33)^ todas as parturientes são testadas para HIV e Sífilis no momento do parto e, quando positivas, seus neonatos também são avaliados. A partir dos resultados positivos para sífilis, a díade mãe e lactente é inserida em tratamento perinatal por 10 dias antes da alta hospitalar. Assim, a ausência de alterações audiológicas observadas neste estudo de caso confirma os efeitos positivos dessa Política Pública no Brasil.

Por outro lado, as alterações auditivas na sífilis congênita não necessariamente se manifestam nos primeiros meses de vida, podendo surgir ao longo da infância e na vida adulta^(6)^. Portanto, é fundamental estabelecer um acompanhamento semestral para lactentes afetados pela sífilis congênita, a fim de identificar o mais precocemente possível, alterações auditivas e de desenvolvimento^(6)^.

A investigação da via auditiva de forma mais abrangente, assim como a ativação cortical para estímulos de fala é relevante nessa população^(7)^, particularmente em casos com outros comprometimentos, como a neurossífilis, em virtude da infecção bacteriana atingir o sistema nervoso central^(6)^. Em acometimentos que podem causar impactos diversos na infância, torna-se também essencial o acompanhamento do desenvolvimento infantil. Em casos de Sífilis Congênita tratada, foi observado desempenho na escala Bayley-III nos primeiros meses de vida semelhante aos lactentes de grupo controle com a mesma faixa etária, evidenciando que a maioria dos sujeitos teve um desempenho na média ou acima dela nas subescalas da Bayley^(34)^. Contudo, o resultado encontrado no presente relato de caso, chama atenção quanto ao desempenho abaixo da média em linguagem expressiva e motor fino, e limítrofe em cognição e linguagem receptiva, sendo necessário guardar as devidas proporções por se tratar de um caso de neurossífilis, indicando a necessidade de orientação à família e seguimento.

Nesse momento de avaliação aos 4 meses, observou-se um impacto do desenvolvimento da lactente, no entanto em virtude do desenho do relato de caso torna-se desafiadora a conclusão se esse impacto se refere diretamente a infecção da sífilis, ou estaria também relacionado à outros indicadores perinatais como a hiperbilirrubinemia ou a questões socioeconômicas.

Quanto às respostas do fNIRS com estímulo de fala pode ser observado neste caso clínico que houve mudança na hemodinâmica cerebral caracterizada pela diferença de fase e a correlação temporal entre as séries temporais de HbO e HbR^(35)^ tanto na presença dos estímulos /ba/ quando no /da/, evidenciando a detecção auditiva desta lactente de quatro meses com neurossífilis.

Constatou-se resposta semelhante da HbO de ambos os estímulos com maior valor de *theta* no ROI anterior de ambos os hemisférios e também no ROI medial do HD. Os canais com maior amplitude no valor de theta foram os da região anterior do lobo temporal de ambos os hemisférios. No caso descrito a lactente não teve nenhuma diferença entre as respostas observadas entre os estímulos, mas evidencia-se que a mesma detectou os sons de fala.

No caso clínico apresentado não houve lateralização da resposta para o hemisfério esquerdo o que se distingue de outros estudos^(16,36)^, que evidenciam uma maior lateralização das respostas para o hemisfério esquerdo. Além disso, observa-se uma ativação predominantemente anterior do lobo temporal e bilateral encontrada neste relato de caso que também se diferencia das pesquisas que demonstraram uma maior extensão de ativação na região cortical temporal do cérebro.

No entanto, é importante destacar que esses estudos foram conduzidos em populações sem indicadores de risco para deficiência auditiva^(19,36-39)^. Portanto, é possível inferir que os resultados do fNIRS encontrados neste caso podem estar relacionados ao acometimento da neurossífilis, assim como os impactos de neurodesenvolvimento observados. Porém, é necessário o estudo de novos casos clínicos para determinar se o padrão constatado neste relato de caso pode ser o padrão relacionado a neurossífilis ou a uma variação das respostas típicas, visto que ainda são incipientes os estudos com fNIRS nesta população.

O fNIRS pode ser uma importante ferramenta para esse acompanhamento da organização funcional do cérebro infantil durante o desenvolvimento, pois mede a atenuação da luz pelos tecidos cerebrais em virtude das alterações na concentração de hemoglobina oxigenada (HbO) e hemoglobina desoxigenada (HbR) dentro da região registrada. Tais alterações refletem a resposta vascular associada à atividade neural e, desta forma, representam uma medida da ativação funcional do cérebro^(35)^. A conectividade funcional mesmo em estado de repouso como no registro realizado do fNIRS com estímulos de fala neste relato de caso, que a lactente estava em sono natural, pode ser observada pela organização funcional intrínseca do cérebro^(35)^. Portanto, as mudanças observadas na HbO e HbR durante a presença e ausência dos estímulos auditivos durante o sono evidenciam que houve resposta de detecção auditiva.

Destaca-se como limitações desta pesquisa, o fato de ser um estudo de caso único, visto que de modo geral, as técnicas de neuroimagem em populações em desenvolvimento podem ter influências e variações como a presença de artefatos de movimento, durações curtas de registro e pequeno tamanho de amostra o que dificulta a generalização de padrões esperados por faixa etária neste tipo de procedimento^(19)^.

Assim, o relato de caso apresentado ilustra um padrão de resultado para o qual será necessária a continuidade de acompanhamento para identificar se as alterações do desenvolvimento ainda ocorrerão em avaliações posteriores e se as mesmas se correlacionam com o padrão das respostas de neuroimagem (fNIRS).

A impossibilidade de acompanhamento longitudinal da criança, uma vez que a família tem várias questões de vulnerabilidade social e apresenta dificuldades importantes para se deslocar para as reavaliações presenciais tanto médicas quanto audiológicas e do neurodesenvolvimento, reduziu a possibilidade da observação de seguimento, que sabe-se, necessária, para esta população.

Em virtude da vulnerabilidade familiar, entende-se que a criança possa estar exposta a outros Determinantes Sociais da Saúde que também podem interferir no seu curso de desenvolvimento, sendo mais um fator que reforça a necessidade desse acompanhamento/monitoramento. Esta é outra questão frequentemente relacionada à sífilis em gestantes e evidenciado nas possíveis consequências geradas na sífilis congênita, incluindo a neurossífilis. Estudo na região de Tocantins^(12)^ evidenciou 29 casos de neonatos com neurossífilis dos quais a maioria era assintomática e com nível socioeconômico baixo e menor escolaridade materna.

Há evidências na literatura que apontam atraso no desenvolvimento neuropsicomotor em crianças com sífilis congênita e neurossífilis^(6,9,10)^. No que se refere aos aspectos audiológicos, além do acompanhamento na atenção especializada na rede de atenção à saúde auditiva, previsto nas diretrizes internacionais e nacionais^(11,40)^, os aspectos de desenvolvimento devem ser acompanhados na Atenção Primária à Saúde, com encaminhamento para avaliação especializada quando identificada a necessidade^(40)^. Quanto aos outros domínios do desenvolvimento, em especial, cognitivo, de linguagem e motor, este caso evidenciou a possibilidade destes impactos que devem ser observados ao longo do desenvolvimento destes lactentes para identificação e intervenção precoces. Assim, a partir do diagnóstico de neurossífilis, essa vigilância e integração em todos os níveis de atenção à saúde torna-se ainda mais urgente, sendo essencial o acompanhamento multidisciplinar.

É necessário que seja ampliado o estudo da interação da sífilis com as possíveis alterações do neurodesenvolvimento, prevendo assim, atrasos no desenvolvimento de linguagem e cognitivo destas, aprimorando, desta forma, as medidas de identificação, diagnóstico e intervenção precoce de alterações no neurodesenvolvimento destes lactentes, contribuindo também, para o aprimoramento das políticas públicas relacionadas ao seguimento na Rede de Cuidado Materno-Infantil nos primeiros anos de vida.

## CONCLUSÃO

A descrição deste caso clínico evidenciou mudança na hemodinâmica cerebral na presença de estímulos de fala sendo caracterizada por maior ativação na região anterior do lobo temporal de ambos os hemisférios cerebrais. Constatou-se atraso no desenvolvimento dos domínios motor e da linguagem expressiva. Estes achados enfatizam a importância da Rede de Cuidado Integral de lactentes com sífilis congênita envolver o monitoramento audiológico e o desenvolvimento infantil.
